# A Case report of a continuous ambulatory drug delivery (CADD) pump to deliver opioid agonist treatment in an acute care setting

**DOI:** 10.1186/s12954-023-00813-x

**Published:** 2023-09-04

**Authors:** Casey Wigg, Amy Nolen, Lisa Jauhal, Malika Sharma

**Affiliations:** 1 Sunnybrook Health Science Centre, 2075 Bayview Ave, Room H266, Toronto, ON M4N 3M5 Canada; 2 Sunnybrook Health Science Centre, 2075 Bayview Ave, Room H336, Toronto, ON M4N 3M5 Canada; 3 Women’s College Hospital, Substance Use Service, 76 Grenville Street, 3rd floor, Toronto, ON M5S 1B2 Canada; 4 Staff Physician and Education Lead, Division of Infectious Diseases, St. Michael’s Hospital, 30 Bond Street, 4-179 CC North, Toronto, ON M5B 1W8 Canada

**Keywords:** Harm reduction, Retention in care, Injectable opioid agonist, Substance use disorder

## Abstract

**Background:**

People with substance use disorder are at risk of complications of drug use and frequent hospitalization and may continue to use substances during admission to acute care. Acute care harm reduction strategies including oral or injectable prescription opioids may aid in care retention and improve health outcomes in this patient population.

**Case presentation:**

A 58-year-old woman with refractory opioid use disorder was admitted to hospital for management of dysphagia secondary to esophageal stricture. She received injectable opioid agonist therapy using a continuous ambulatory drug delivery (CADD) pump in order to facilitate completion of her hospital admission.

**Conclusions:**

The patient successfully received acute medical care with the use of a CADD pump for consistent, patient-controlled opioid administration, with the support of an interdisciplinary team and by respecting the patient’s own substance use goals.

## Introduction

Harm reduction is a patient centered, evidence-based approach compromising one aspect of care for people with substance use disorder (SUD) [1]. Harm reduction does not require abstinence from substance use; rather it is meant to reduce the potential health and social harms associated with drug use [2–4]. Despite the availability of opioid agonist therapy (OAT) such as buprenorphine/naloxone (suboxone), methadone and naltrexone, a proportion of hospitalized patients may not respond, have access to or be accepting of these interventions for substance use [5]. Stigma from healthcare staff, hospital policies enforcing abstinence, suboptimal pain management, and withdrawal symptoms can often result in involuntary or patient-directed discharge, thereby driving poor health outcomes [6].

In Canada, harm reduction services such as distribution of sterile equipment, safe disposal of used equipment, supervised consumption services and distribution of overdose prevention kits are readily utilized in the community setting and not widely implemented in inpatient settings [7. In acute care settings, people with SUD are at risk of complications of substance use, as well as the risk of patient-directed discharge should they need to leave the hospital in order to use [1]. The implementation of harm reduction strategies in the acute care setting may mitigate negative health outcomes [6, 8]. There is evidence that offering prescription injectable hydromorphone or diacetylmorphine (pharmaceutical-grade heroin) in the community setting for people with severe, refractory opioid use disorder improves health and social outcomes [9, 10]. Similarly, prescription opioids (oral and injectable) have been proposed as a treatment option in the acute care environment for patients with severe opioid use disorder [7, 11]. We report a case of a female patient in acute care with a longstanding opioid use disorder who received injectable opioid agonist therapy using a continuous ambulatory drug delivery (CADD) pump in order to facilitate completion of her hospital admission.

A CADD pump contains a reservoir of medication, typically a parenteral opioid such as morphine, hydromorphone or fentanyl, and can be programmed to administer an automated hourly infusion of medication through an intravenous or subcutaneous line. The pump can also deliver patient-initiated bolus doses at pre-programmed time intervals, with a lockout period to prevent inadvertent toxicity. CADD pumps are typically reserved for the management of acute pain in the post-operative period, or for management of chronic malignant pain or dyspnea in patients with life-limiting diagnoses. Utilizing a CADD pump for the purposes of reducing harm by providing a safer opioid supply was uncharted territory for our team.

### Case presentation

A 58-year-old woman with a 30-year history of infection with human immunodeficiency virus (HIV) was admitted to hospital for management of dysphagia secondary to esophageal stricture. The patient was diagnosed with a squamous cell carcinoma of the oropharynx 2 years prior and received treatment with an extended course of radiation therapy. She remained free of cancer recurrence but developed esophageal stricture as a late complication of radiation therapy. Her past medical history was complex; in addition to HIV infection, she was known to have HIV-induced immune thrombocytopenic purpura, hepatitis C infection, chronic obstructive pulmonary disease, depression, recurrent facial cellulitis, and polysubstance use including opioid use disorder. Her esophageal stricture resulted in progressive dysphagia, and she was eventually unable to swallow any tablets including her anti-retroviral therapy.

She had been using injection drugs for several decades, including heroin, crack cocaine, and fentanyl. She was prescribed slow-release oral morphine (SROM) (60 mg three times a day) and immediate release oral morphine (10 mg four times a day) by her community addiction specialist as opioid agonist therapy (OAT). As the patient’s dysphagia progressed, she began to crush, heat, and inject her prescribed opioids. At the time of admission to hospital she also reported using crack cocaine 10–12 times per month.

Historically, the patient preferred to receive medical management in the outpatient setting. When hospital admission was required, she would often leave before treatment completion against medical recommendations. During previous hospitalizations, the patient experienced stigma surrounding her substance use and expressed frustration with practitioners who under dosed or delayed the administration of opioids because of her substance use history, thereby prompting patient-directed discharge.

The patient presented to her outpatient HIV clinic dehydrated and cachectic secondary to severe dysphagia. Her facial cellulitis caused periorbital edema resulting in extremely limited vision; despite this she was injecting her prescribed morphine with varied success. Outpatient care options had been exhausted and she required an urgent hospital admission for lifesaving therapies. In order to engage the patient with inpatient care, the team proposed a CADD pump to deliver opioids at a dose equivalent to her community prescription, and to offer the option of patient-controlled breakthrough dosing thus returning control to the patient. She agreed to present to the emergency department, with the hope that a CADD pump would alleviate the constant need to advocate for OAT in acute care.

### Clinical findings and timeline

The patient was forthcoming about her opioid use and transparent about her plan to continue using opioids in the community. The team recognized that enforcing abstinence or OAT with buprenorphine-naloxone or methadone would deter her from engaging with care. Increasing SROM was not an option because of her severe dysphagia, and hospital policy restricted the delivery of IV opioids on the unit thus removing the option of injectable (intravenous) hydromorphone. Instead, a shared decision was made to modify risk through subcutaneous opioid agonist therapy delivered through a continuous ambulatory drug delivery (CADD) pump. A CADD pump provided her with the autonomy to self-administer breakthrough doses without having to rely on staff.

The patient’s home morphine dose was converted to the parenteral equivalent, which proved challenging as the precise bioavailability of sustained release oral morphine when injected is unknown. Further, because of her impaired vision, her degree of success in injecting her opioids in the days prior to admission was questionable, thus her true opioid requirements were not clear. During the initial period in the emergency department, the patient was started on intermittent subcutaneous hydromorphone as a bridge until she transferred to the medical unit and could be initiated on a CADD pump. see Table 1 for details of opioids prescribed.

**Table 1:** Substances and dosing at presentation to hospital and during admission

Location/timeline	Substance and dosing	Variables considered	Lessons learned
Community (at time of presentation to hospital)	Crack cocaine 10–12 times per month SROM^*^ 60 mg three times per day Morphine IR^**^ 10 mg four times per day 24-h total morphine: 220 mg oral daily equivalents (+ crack cocaine)	Patient had impaired mobility from generalized weakness and had not been able to access crack cocaine in the period prior to admission. She may have been experiencing withdrawal symptoms from crack on arrival to hospital	We overlooked the possibility that her community morphine dose had not stabilized her opioid needs and thus she was simultaneously using crack cocaine
Emergency Department	Initially Hydromorphone 1.5 mg sc q3hr 20 h later, rotated to morphine 8 mg sc q3h with no PRN dosing ordered	20 h after admission, patient was described as “irritated and upset with pain control” Palliative Care was consulted to optimize pain medication	In the first 24 h, patient received a reduced morphine equivalent dose from 220 mg to 24 h in community to 128 mg to 24 h. She became increasingly agitated with no signs of overdose. Consideration of PRN dosing may have prevented withdrawal
Acute care days 1–2	24 h into admission, Subcutaneous morphine 3 mg/hr continuous infusion with breakthrough dose of 2 mg every one hour PRN via CADD pump 24-h total morphine: ~ 140 mg oral daily equivalents	Although prescribed SROM in the community, the patient was crushing, heating and injecting her opioids (because she was unable to swallow tablets), thus converting opioids into an immediate release formulation It was unclear to what extent she was successfully injecting due to her impaired vision The precise bioavailability of sustained release oral morphine when injected is unknown	The patient was new to the team and there were questions about exact dosing in the community; however, the patient was clearly presenting with signs of withdrawal Morphine equivalent dose of 140 mg/24 h was an increase from what she received in the first 24 h in ER; however, there was still an 80 mg discrepancy from her community dose In retrospect there was an abundance of caution in watching for oversedation and insufficient attention to the discrepancy leading to withdrawal symptoms
Acute Care Day 3	Subcutaneous morphine 5 mg/hr continuous infusion with breakthrough dose 5 mg every hour PRN via CADD pump) In addition to the CADD pump, Morphine 15 mg subcutaneous QID while titrating CADD pump to manage withdrawal symptoms Lorazepam 1 mg every 4 h PRN for agitation with withdrawal	On Day 3 of admission, the patient was experiencing withdrawal symptoms (agitated, restless, muscle twitches and insomnia) and it was evident that her baseline opioid coverage was insufficient	More rapid uptitration was warranted to address withdrawal symptoms
Acute Care Day 4	ADD pump basal rate was discontinued and the bolus rate increased to 5 mg every one hour PRN. Morphine 15 mg subcutaneous QID PRN was continued. Ativan was decreased to 1 mg TID PRN	Patient left AMA^α^ overnight and returned the next morning. She presented as drowsy, coherent at times but predominantly slurring words Esophageal stricture was treated with dilation later in day	The patient did not acknowledge using a substance in the community. The patient was monitored for oversedation and, however, was continued on morphine to prevent another episode of withdrawal
Acute Care Day 5 until discharge on Day 21	Post-esophageal dilatation, she was transitioned to SROM 60 mg TID CADD pump remained in situ for breakthrough 8 mg morphine every hour as needed	Once patient was able to tolerate oral medications, morphine was provided orally but the CADD pump remained for breakthrough dosing	The patient did not display any further signs of withdrawal and remained engaged with care. She typically used the breakthrough dose available and had numerous additional attempts. The CADD pump provided the patient with autonomy and avoided the numerous requests for breakthrough dosing and decreased the nursing workload

*SROM = sustained release oral morphine

**IR = immediate release oral morphine

α = against medical advice

Despite having a team committed to providing harm reduction, on Day 3 of admission the patient experienced withdrawal symptoms and it became evident that her baseline opioid coverage was insufficient. Over the subsequent two days, the patient was assessed multiple times per day and her CADD dosing was uptitrated to prevent withdrawal and cravings. On Day 5 of admission, she underwent gastroscopy with dilatation of her esophageal strictures. The subsequent day, she was cleared by the speech language pathologist to resume oral intake. The hourly rate on the CADD pump was stopped and she was able to resume her home dose of SROM by mouth. She continued the CADD for bolus dosing of morphine until she was medically stable for transfer to a transitional facility for ongoing supportive care. She remained stable on her home dose of SROM during transition care.

### Follow-up and outcomes

Over the subsequent months, the patient’s esophageal strictures recurred, and she required readmission on several occasions to optimize nutrition and for eventual feeding gastrostomy tube insertion and tracheostomy. Her opioid use disorder was managed with the CADD pump on each presentation. With every admission, she was more willing to engage with the healthcare system with the knowledge that she would receive effective treatment with reduced fear of discrimination and stigmatization.

### Informed consent

Written informed consent from the patient, Katherine McNeil, was obtained to report her case in the medical literature. Katherine specifically requested use of her real name. Katherine was a brave and generous woman with goals and dreams, despite facing tremendous challenges and tragedy. The months she spent in the hospital were difficult and working closely on the manuscript with the authors provided her a sense of hope and purpose.

## Discussion

We describe a case of a woman with severe opioid use disorder who received injectable opioid agonist therapy using a continuous ambulatory drug delivery (CADD) pump in order to facilitate completion of a hospital admission. This unconventional harm reduction strategy was borne out of the requirement to support a patient in need of medical intervention but with continued use of substances.

This approach succeeded in engaging the patient in care for several reasons. Firstly, in acute care we were well-positioned to conduct routine assessments for any withdrawal or oversedation symptoms (using continuous oximetry). In the past, when the patient displayed irritability and frustration toward staff, this was dismissed as the ‘drug-seeking behavior of a challenging patient.’ We recognized the behavior as a coping mechanism in a patient experiencing withdrawal, and the symptoms were mitigated with regular monitoring and rapid uptitration of injectable OAT (iOAT) via the CADD pump. Notably, this is a resource-intensive process that requires appropriate training of staff; however, by taking advantage of the controlled, high-resourced environment in the acute care setting optimal patient care was delivered.

The patient was also supported by an interdisciplinary approach to her care. She had a history of negative interactions with the medical establishment and often felt misunderstood and judged in hospital settings. Her long-standing nurse practitioner from the HIV clinic advocated daily for her care needs. Marginalized or ‘challenging’ patients benefit when they have an advocate who can remind the team of their humanity. Addiction and palliative care specialists collaborated to manage the CADD pump and advocated to use a CADD pump as a novel approach to accessing iOAT in hospital. The team’s social worker facilitated the transition to a step-down hospital that could support the patient’s goals of care. Her community addiction specialist was in close communication with the hospital team to ensure a seamless discharge back to community-based care.

Most critically, the medical team placed the patient’s own goals at the center of her care plan. The primary goal of her admission was for definitive management of her dysphagia; she did not voice a desire for drug rehabilitation or abstinence and it is crucial that patients are not faced with an ultimatum in order to receive essential health care services. Individuals with opioid use disorder face stigma in hospital, and this is further exacerbated by punitive responses from a medical establishment that perpetuate power imbalances for marginalized patients. The CADD pump was successful in part because it returned a degree of control to the patient, thereby empowering her to achieve her individualized treatment goal.

Admittedly, there are potential barriers and obstacles to using a CADD pump for harm reduction purposes. Although it provides a safer opioid supply and allows the patient control over breakthrough doses, it can be time consuming for staff particularly during the initial stage of titrating and monitoring. However, once a therapeutic dose is established, nurses no longer need to provide regular breakthrough doses thereby significantly reducing. The additional cost and physical resource of having CADD pumps available could prohibit uptake of CADD pumps for harm reduction purposes. Lastly, there is expertise and knowledge required in prescribing and programming a CADD pump.

The patient successfully received acute medical care with the use of a CADD pump for consistent, patient-controlled opioid administration, with the support of team-based collaborative care and by respecting the patient’s goals. To replicate this success and establish a strong foundation for harm reduction interventions, we advocate for system-level changes including mandatory educational programs to enable de-stigmatizing care, as well as the creation of harm reduction committees to identify champions within healthcare institutions to spearhead evidence-based, non-coercive policies for people with SUD in acute care. Formal policy change, educational and accreditation requirements will help to create hospitals that are safe for all people to make healthy choices based on their individual needs.

## Data Availability

Not applicable.

## Abbreviations

AMA: Against medical advice
CADD: Continuous ambulatory drug delivery
HIV: Human immunodeficiency virus
iOAT: Injectable OAT
IR: Immediate release oral morphine
OAT: Opioid agonist therapy
SROM: Slow-release oral morphine
SC: Subcutaneous
SUD: Substance use disorder

## References

[1] Dong KA, Brouwer J, Johnston C, Hyshka E (2020). Supervised consumption services for acute care hospital patients. CMAJ.

[2] Harm Reduction. https://ontario.cmha.ca/harm-reduction/.Accessed 30 Mar 2023

[3] Bruneau J, Ahamad K, Goyer MÈ (2018). Management of opioid use disorders: a national clinical practice guideline. CMAJ.

[4] Ontario Drug Policy Research Network T. On behalf of: changing circumstances surrounding opioid-related deaths in Ontario during the COVID-19 pandemic. Published online 2021.

[5] Fairbairn N, Ross J, Trew M (2019). Injectable opioid agonist treatment for opioid use disorder: a national clinical guideline. CMAJ.

[6] Sharma M, Lamba W, Cauderella A, Guimond TH, Bayoumi AM (2017). Harm reduction in hospitals. Harm Reduct J.

[7] Guidance Document on the Management of Substance Use in Acute Care. Published January 2020, Alberta. Available at: https://crismprairies.ca/management-of-substance-use-in-acute-care-settings-in-alberta-guidance-document/.. Accessed 30 Mar 2023.

[8] Dong K, Meador K, Hyshka E, et al. Supporting people who use substances in acute care settings during the COVID-19 pandemic: CRISM - interim guidance document. Edmonton, Alberta: Canadian Research Initiative in Substance Misuse; 2020. p.62. Version 2.

[9] Oviedo-Joekes E, Guh D, Brissette S (2016). Hydromorphone compared with diacetylmorphine for long-term opioid dependence: a randomized clinical trial. JAMA Psychiat.

[10] Maghsoudi N, Bowles J, Werb D (2020). Expanding access to diacetylmorphine and hydromorphone for people who use opioids in Canada. Can J Public Health.

[11] Eydt E, Glegg S, Sutherland C (2021). Service delivery models for injectable opioid agonist treatment in Canada: 2 sequential environmental scans. Can Med Assoc Open Access J.

